# Genetic Variants With Small Discordant Effects on eGFR and Risk of CKD

**DOI:** 10.1016/j.ekir.2026.107011

**Published:** 2026-08-13

**Authors:** Archish Sadeesh, Pukhraj Gaheer, Nikhil Uppal, Matthew B. Lanktree

**Affiliations:** 1 Faculty of Engineering, Integrated Biomedical Engineering and Health Sciences, McMaster University, Hamilton, Ontario, Canada; 2 Population Health Research Institute, Hamilton, Ontario, Canada; 3 Department of Health Research Methods, Evidence & Impact, McMaster University, Hamilton, Ontario, Canada; 4 Division of Nephrology, St. Joseph’s Healthcare Hamilton, Hamilton, Ontario, Canada; 5 Department of Medicine, McMaster University, Hamilton, Ontario, Canada

**Keywords:** CKD, eGFR, genomics

## Introduction

Chronic Kidney Disease (CKD) is defined by sustained reductions in estimated glomerular filtration rate (eGFR) and the presence of albuminuria. A single eGFR assessment—derived from creatinine or cystatin C—fail to capture glomerular hyperfiltration, a state where increased clearance per nephron imposes mechanical or metabolic stress. Because we lack reliable methods to estimate nephron number or mass, the essential denominator to determine single-nephron GFR, hyperfiltration remains a “silent” early maladaptive response.^1^ Hyperfiltration arises through afferent arteriolar vasodilation, renin-angiotensin-aldosterone system activation, and increased intraglomerular pressure, driving podocyte stretch; mesangial expansion; and progressive nephron loss. Experimental and human studies in diabetes support that hyperfiltration precedes albuminuria and triggers molecular signatures of podocyte and tubular epithelial stress. Obesity and nephron loss models similarly show compensatory increases in single nephron GFR coupled to glomerular hypertension and cytoskeletal remodeling. We hypothesized that genetic variants associated with higher cross-sectional eGFR but increased long-term CKD risk may reflect genetically mediated hyperfiltration, revealing biological pathways distinct from those captured by conventional CKD definitions.

Although the physiological and cellular consequences of hyperfiltration are well defined, its genetic determinants remain unexplored, primarily because there is no scalable measure of hyperfiltration. Genome-wide association studies (GWAS) of eGFR are largely focused on individuals enrolled in population-based cohorts with eGFRs in the normal range and often only cross-sectionally.^2^^,^^3^ eGFR decline can be defined in different ways including simple deltas or using linear mixed models, with pros and cons of each.^4^ GWAS of longitudinal decline in eGFR have had fewer novel loci than cross-sectional studies because of a mix of statistical and power issues, with generally smaller samples.^5^^,^^6^ GWAS of dichotomous CKD definitions have used an eGFR threshold of 60 ml/min per 1.73 m^2^ (CKD stage G3a) representing relatively early-stage CKD, with only a small proportion progressing to kidney failure.^3^

Comparing the genetic architecture of eGFR and CKD definitions seems, on the surface, like a tautology—a circular or redundant analysis—given that CKD is defined by levels of eGFR. A similar paradigm exists in endocrinology, where studies have examined genetic variants associated with fasting glucose, hemoglobin A1C, and diagnosed type 2 diabetes, with studies of each highlighting distinct biological signatures and architectures.^7^ Is it reasonable to hypothesize that genetic variants associated with a lower cross-sectional eGFR in a normal population are associated with higher risk of CKD? Could genetic effects vary with time? Could a variant be associated with higher eGFR early in disease progression, representing hyperfiltration, that is later associated with kidney failure in the long run? We hypothesized that variants with directionally discordant effects on cross-sectional eGFR and CKD risk may identify candidate loci relevant to mechanisms such as nephron endowment or hyperfiltration. We therefore compared per-allele eGFR and CKD effect estimates and annotated the resulting regional signals to nearby genes.

## Results

Using data from published GWAS, ∼ 8.8 million harmonized variants were examined, and after filtering for genome-wide significance with eGFR, ∼ 60,000 variants were retained.^4^^,^^5^ The effects of each allele on each trait were normalized against the effects of all observed variants (Supplementary Methods). A clear correlation exists between the genetic effects on eGFR and CKD—alleles associated with higher eGFR are, on average, associated with lower CKD risk and vice versa (Figure 1). Variants with residuals (i.e., the distance from the fitted regression line representing the correlation of effects on eGFR and CKD risk) > 2 standard deviations were further examined. Plotting discordant points by their genomic position and the degree of discordance (i.e., the residual) revealed peaks reflecting clusters of variants in loci with discordant signals, suggesting biologically coherent signals rather than stochastic noise (Figure 2).

**Figure 1:**
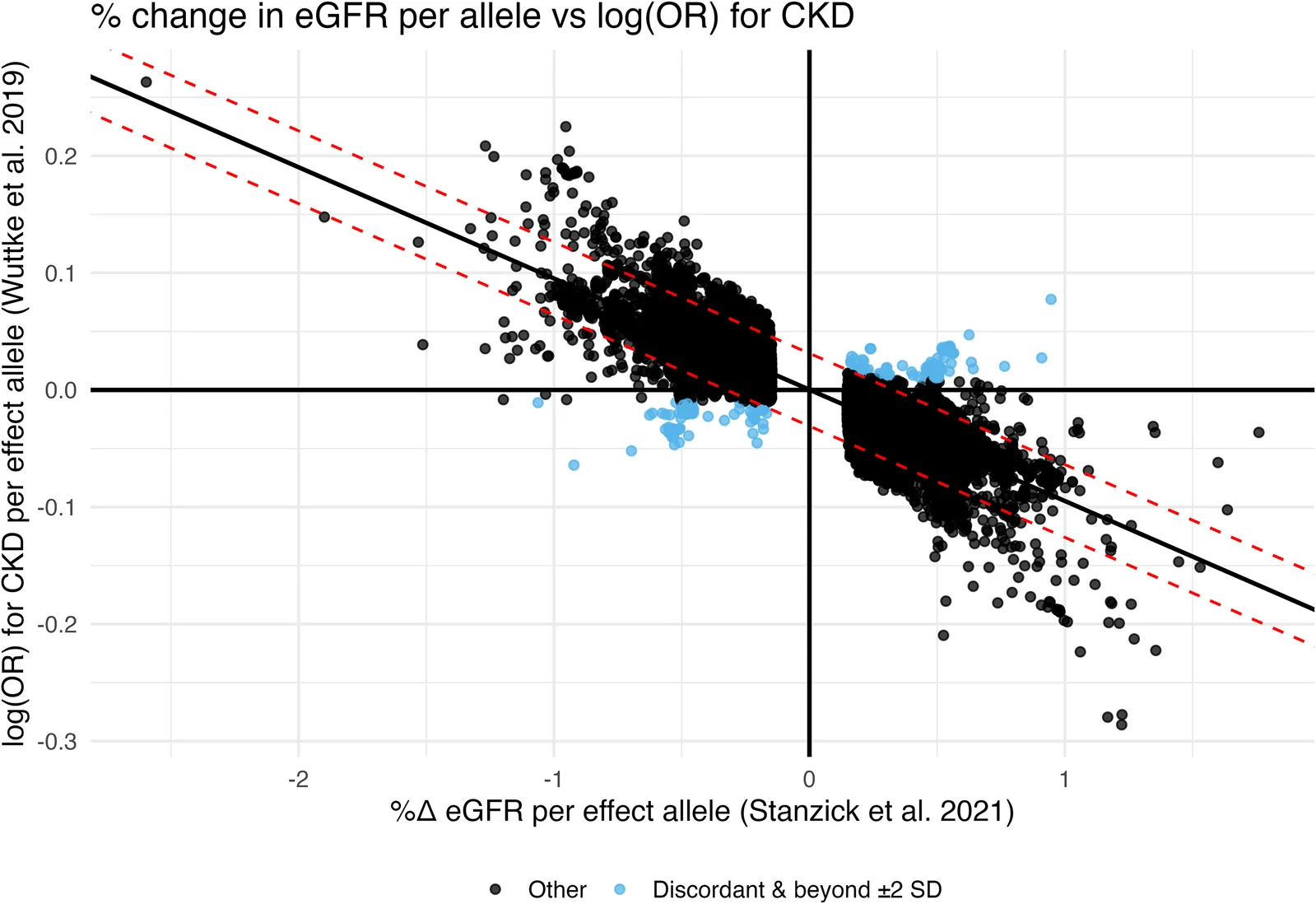
Relationship between genetic effects on cross-sectional eGFR and CKD risk. The percentage change in eGFR per effect allele is plotted against the CKD log-odds estimate for the same harmonized effect allele. The solid line represents the fitted regression, and the dashed lines represent ±2 standard deviations of the residual distribution. Blue points are directionally discordant variants beyond the residual threshold that also met the minimum-effect criteria; black points are all remaining eGFR-associated variants. Labels indicate the nearest annotated genes for selected lead variants. CKD, chronic kidney disease; eGFR, estimated glomerular filtration rate.

**Figure 2:**
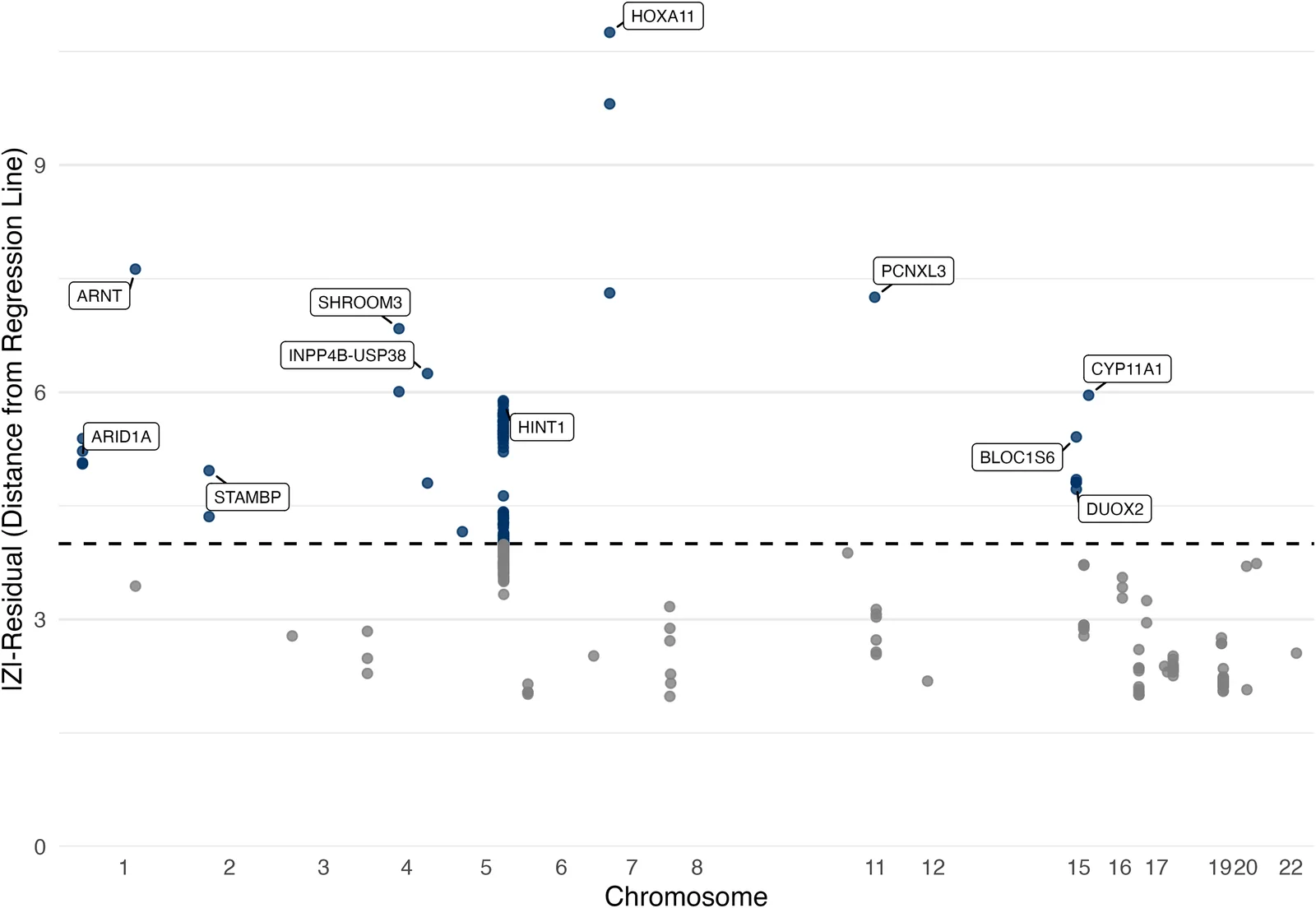
Genomic position of variants with discordant effects on eGFR and CKD risk. Candidate variants are plotted by chromosomal position against the absolute standardized residual from the primary regression. Gray points have (2<|Z|<4), and blue points have (|Z|≥4). The dotted horizontal line at (|Z|=4) is provided for visual emphasis and was not used to define the complete candidate set. Labels indicate the nearest annotated genes for selected lead variants. CKD, chronic kidney disease; eGFR, estimated glomerular filtration rate.

A total of 319 variants were clustered in 11 genetic loci with discordant effects on eGFR and CKD risk (lead variants for each locus in Supplementary Table S1). Clusters of variants are because of linkage disequilibrium between variants adjacent to each other in the genome and multiple variants in a cluster increase the likelihood that it is a true signal (LocusZoom plots in Supplementary Figures S1–11). Given that variants were selected for an association with eGFR, all genes have established relevance to kidney function. Those with discordant effects on eGFR and CKD included the *HOXA11* region (implicated in congenital anomalies of the kidney and urinary tract), *ARNT* (hypoxia-responsive transcriptional program linked to antifibrotic and proregenerative signaling), and *SHROOM3* (essential for nephron development).

Inverse-variance weighting produced a similar regression slope to the primary analysis (−0.097 vs. −0.095) and identified 173 candidate variants. Of these, 148 (86%) overlapped the primary candidate set, and 7 of the 11 prespecified lead variants retained candidate status.

## Discussion

Most variants display a concordant direction of effect on cross-sectional eGFR and risk of CKD (defined as an eGFR of less than 60 ml/min per 1.73 m^2^). However, we identified a subset of variants with unexpectedly discordant effects. These discordant variants clustered in 11 genomic loci and highlight genes spanning developmental determinants of nephron mass, filtration barrier integrity, tubular injury signaling, and fibrosis.

The *HOXA11* and *SHROOM3* loci provide plausible developmental hypotheses involving nephron endowment, whereas *ARNT* may implicate pathways involved in chronic kidney injury responses. The *HINT1* locus contained the densest accumulation of candidate variants, although this density reflects correlated variants in linkage disequilibrium rather than multiple independent signals. Furthermore, the regional annotations are proximity-based, and additional fine-mapping and functional evidence are required to pin down the causal genes and mechanisms leading to discordance.

Iterative GWASs of cross-sectional eGFR have increased in sample size to millions of participants, yielding more and more genes with associations with cross-sectional eGFR.^2^^,^^3^ Nevertheless, kidney genetics has been somewhat behind other complex genetic traits, such as diabetes, lipids, and obesity, that received greater attention earlier in the development of GWAS. Different architectures of various glucose traits, such as fasting glucose, insulin concentration and resistance, hemoglobin A1C, and risk of diabetes, have been recognized.^7^ Identification of differences in the genetic architecture for disease diagnosis and disease prognosis is not limited to kidney disease—a study of 9 common diseases found limited overlap between genetic effects on disease diagnosis and genetic effects on progression to disease specific mortality.^8^ Although there has been a required focus to improve and diversify ancestral representation among genetic study participants, GWAS of people with advanced stages of CKD (i.e., eGFR < 15 ml/min per 1.73 m^2^) are still significantly underrepresented and lacking from the literature. Partially because of the success of exome sequencing studies searching for rare variants with large effects (also known as monogenic or Mendelian CKD), GWAS of advanced CKD has not yet been done at large scale. Variants impacting hyperfiltration, inflammation, or fibrosis would not be expected to be identified in GWAS of eGFR in healthy people in population scale data banks.

In multi-trait GWAS, the power to detect important variants is improved by integrating their effects on multiple traits, which is related but a separate approach to the current study.^9^ Challenges in measuring, modelling, and quantifying longitudinal change in eGFR include the number of measurements, how to handle missingness and variable follow-up, the statistical methods, and the need for access to individual-level data.^4^ Moreover, when evaluating the effect of variants only among people with CKD, there is the possibility of collider bias—those with greater traditional risk factors may paradoxically have lower genetic risk factors. However, this report should encourage genetic studies into the cumulative impact of common small effect variants on CKD, especially as we shift into the era of whole genome sequencing where these common intronic or intergenic variants are collected.

This analysis did not directly measure hyperfiltration, nephron number, single-nephron GFR, albuminuria, longitudinal eGFR decline, or kidney failure. The identified variants should be considered candidate discordant loci rather than evidence of hyperfiltration-causing variants. Individual CKD effect estimates are imprecise, although the overall relationship remained robust in inverse-variance-weighted sensitivity analysis. The analysis was restricted to available European-ancestry summary statistics, and causal genes cannot be assigned from proximity annotation alone.

In summary, we identified a set of variants with discordant effects on eGFR and CKD risk that are plausibly related to lower nephron mass, hyperfiltration, and fibrosis. Variants impacting kidney disease may not be uncovered at all while examining individuals with eGFR in the normal range. Although “GWAS fatigue” exists, collection of genome-wide genotyping data, whether from genotyping arrays or whole genome sequencing, and linkage to clinical, biochemical, and multiomic data in individuals from across the spectrum of eGFRs is still needed.

## Disclosure

MBL has received speaker and advisory fees from Otsuka, GlaxoSmithKline, and Hikma Pharmaceuticals on topics unrelated to this work. All other authors have no conflicts of interest to disclose.

## Supplementary Material

Supplementary Files (pdf) **Supplementary Methods. Figure S1.** Detailed LocusZoom plot for *HOXA11* locus. **Figure S2.** Detailed LocusZoom plot for *ARNT* locus. **Figure S3.** Detailed LocusZoom plot for *PCNX3* locus. **Figure S4.** Detailed LocusZoom plot for *SHROOM3* locus. **Figure S5.** Detailed LocusZoom plot for *INPP4B-USP38* locus. **Figure S6.** Detailed LocusZoom plot for *CYP11A1-SEMA7A* locus. **Figure S7.** Detailed LocusZoom plot for *HINT1* locus. **Figure S8.** Detailed LocusZoom plot for *BLOC1S6* locus. **Figure S9.** Detailed LocusZoom plot for *ARID1A* locus. **Figure S10.** Detailed LocusZoom plot for *STAMBP* locus. **Figure S11.** Detailed LocusZoom plot for *DUOX2* locus. **Table S1.** Independent loci in discordant variant set.

## Footnotes

Supplementary Files (pdf)
**Supplementary Methods**. **Figure S1.** Detailed LocusZoom plot for *HOXA11* locus. **Figure S2.** Detailed LocusZoom plot for *ARNT* locus. **Figure S3.** Detailed LocusZoom plot for *PCNX3* locus. **Figure S4.** Detailed LocusZoom plot for *SHROOM3* locus. **Figure S5.** Detailed LocusZoom plot for *INPP4B-USP38* locus. **Figure S6.** Detailed LocusZoom plot for *CYP11A1-SEMA7A* locus. **Figure S7.** Detailed LocusZoom plot for *HINT1* locus. **Figure S8.** Detailed LocusZoom plot for *BLOC1S6* locus. **Figure S9.** Detailed LocusZoom plot for *ARID1A* locus. **Figure S10.** Detailed LocusZoom plot for *STAMBP* locus. **Figure S11.** Detailed LocusZoom plot for *DUOX2* locus. **Table S1.** Independent loci in discordant variant set.
