# Supplementary material for: Genetic Variants With Small Discordant Effects on eGFR and Risk of CKD
Source: Kidney Int Rep. 2026 Aug 13;11(11):107011. doi: 10.1016/j.ekir.2026.107011 (PMC13634568; doi:10.1016/j.ekir.2026.107011)
Supplement: Supplementary file 1 [file mmc1.pdf]

Supplementary Methods

Data sources and genome build

We analyzed publicly available genome-wide association study (GWAS) summary statistics. Estimated glomerular filtration rate (eGFR, creatinine-based) GWAS effect estimates were obtained from Stanzick et al. (European ancestry meta-analysis, >1 million individuals). Chronic kidney disease (CKD, cases defined by eGFR <60 mL/min/1.73m²) data were obtained from Wuttke et al. (CKDGen, European subset). All genomic coordinates and annotations were mapped to hg19/GRCh37.

Software environment

All analyses were conducted in R. The following packages were used: *TwoSampleMR, coloc.abf, data.table, dplyr, tidyr, ggplot2, ggrepel, plotly, ChIPseeker, TxDb.Hsapiens.UCSC.hg19.knownGene, org.Hs.eg.db, GenomeInfoDb, rtracklayer, purrr, reshape2, ieugwasr*.

Harmonization of GWAS datasets

The eGFR and CKD summary statistics were formatted using the TwoSampleMR package and harmonized using *harmonise_data(action = 2)*. CKD effect estimates were aligned to the same effect allele as the corresponding eGFR estimates, while retaining the original direction of effect; effects were not reoriented to the minor allele or forced to be positive. Allele-frequency information was used to resolve palindromic variants where possible, and variants with unresolved strand ambiguity, mismatched alleles, missing estimates, or non-finite beta values or standard errors were excluded. No proxy variants were used.

Both GWAS were restricted to participants of European ancestry to provide ancestry-matched eGFR and CKD effect estimates and to correspond with the European reference panel used for linkage-disequilibrium estimation. This restriction limits the generalizability of the results to other ancestral populations.

The eGFR estimates were reported on the log scale and converted to percentage change per effect allele as $100 \times [exp(\beta_{eGFR}) - 1]$. CKD estimates were retained on the log-odds scale for regression and exponentiated to odds ratios for presentation.

Residual regression framework

Following harmonization, variants associated with eGFR at genome-wide significance $P < 5 \times 10^{-8}$ were retained, resulting in 58,479 variants. We regressed the per-allele CKD log-odds estimates on the percentage change in eGFR per effect allele. For each variant, the residual was calculated as the observed CKD effect minus the effect predicted by the fitted regression line. Residuals were standardized by dividing each residual by the standard deviation of all model residuals.

Primary candidate variants were required to meet three criteria: (1) directionally discordant effects, defined as either higher eGFR with higher CKD risk or lower eGFR with lower CKD risk; (2) a residual beyond ±2 standard deviations from the fitted line in the discordant direction; and (3) absolute effects of

at least 0.1% change in eGFR and 0.01 CKD log-odds per effect allele. Applying these criteria identified 319 candidate variants.

Manhattan-style residual plot

The genomic distribution of the 319 candidate variants was visualized by plotting their absolute standardized residuals against chromosomal position. The dotted horizontal line at ($|Z|=4$) was used to visually distinguish the most extreme residuals and was not the threshold used to define the complete candidate set. Variants with ($2<|Z|<4$) were displayed in grey and variants with ($|Z|\geq4$) were displayed in blue. Gene labels represent proximity-based annotations for selected lead variants.

Identifying Independent Signals

Independent signals are used to represent clusters of variants in a locus. When multiple variants are in close proximity within a genomic locus, it is difficult to determine which variant is driving the signal at that locus. We calculated the linkage disequilibrium between variants, within the *ieugwasr* package in R. The 1000 Genome European reference panel was used for linkage disequilibrium calculations. Lead variants were selected to be less than an r^2 threshold of 0.1 in a region of greater than 500kb apart. Discordant variants were annotated to nearest or overlapping genes using ANNOVAR. Intergenic, upstream, and downstream annotations were labeled accordingly. Exome capture variants were flagged using ANNOVAR exome outputs.

Inverse-Variance-Weighted Sensitivity Analysis

To account for variation in the precision of CKD effect estimates, we repeated the regression using inverse-variance weights defined as the reciprocal of the squared standard error of each CKD log-odds estimate. Standardized residuals were calculated from the weighted regression model. Weighted candidate variants were required to have directionally discordant effects, a standardized residual beyond ±2 in the discordant direction, an absolute eGFR effect of at least 0.1% per allele, and an absolute CKD effect of at least 0.01 log-odds per allele. Results were compared with the candidate variants and lead variants identified in the primary unweighted analysis.

Supplementary Table S1: Independent loci in discordant variant set.

Gene	Variant (rsID)	C hr	Effect Allele[1]	Other Allele[2]	Freq	eGFR effect[3]	CKD Odds Ratio	eGFR *P* Value	CKD *P* Value	Residual[4]
HOXA11	rs184245386	7	T	C	0.01	0.95	1.08	2.3E-08	0.25	0.16
ARNT	rs188499820	1	T	C	0.98	-0.70	0.95	2.9E-08	0.21	-0.12
PCNXL3	rs200692281	11	A	C	0.99	-1.06	0.99	7.8E-09	0.92	-0.11
SHROOM3	rs114735513	4	T	C	0.02	0.63	1.05	4.8E-08	0.25	0.11
INPP4B-USP38	rs115890303	4	T	C	0.03	-0.53	0.95	4.4E-08	0.27	-0.10
CYP11A1-SEMA7A	rs150562828	15	T	C	0.02	0.76	1.02	1.4E-10	0.59	0.09
HINT1	rs145402536	5	T	C	0.03	-0.54	0.97	9.5E-10	0.17	-0.09
BLOC1S6	rs139334218	15	A	C	0.95	-0.47	0.96	5.7E-11	0.16	-0.08
ARID1A	rs79598313	1	T	C	0.03	0.63	1.02	1.3E-12	0.46	0.08
STAMBP	rs116524817	2	T	C	0.03	0.53	1.03	4.0E-08	0.50	0.08
DUOX2	rs117974015	15	A	G	0.02	-0.58	0.98	6.3E-09	0.64	-0.07

[1]Effect, significance, and allele frequency with respect to eGFR taken from Stanzick et. al, 2021
[2]Other allele refers to the non-effect allele after harmonization of the eGFR and CKD datasets.
[3]% change per allele
[4]Residual distance from regression line, or discordance between expected effects on eGFR and CKD (CKD log-odds scale)

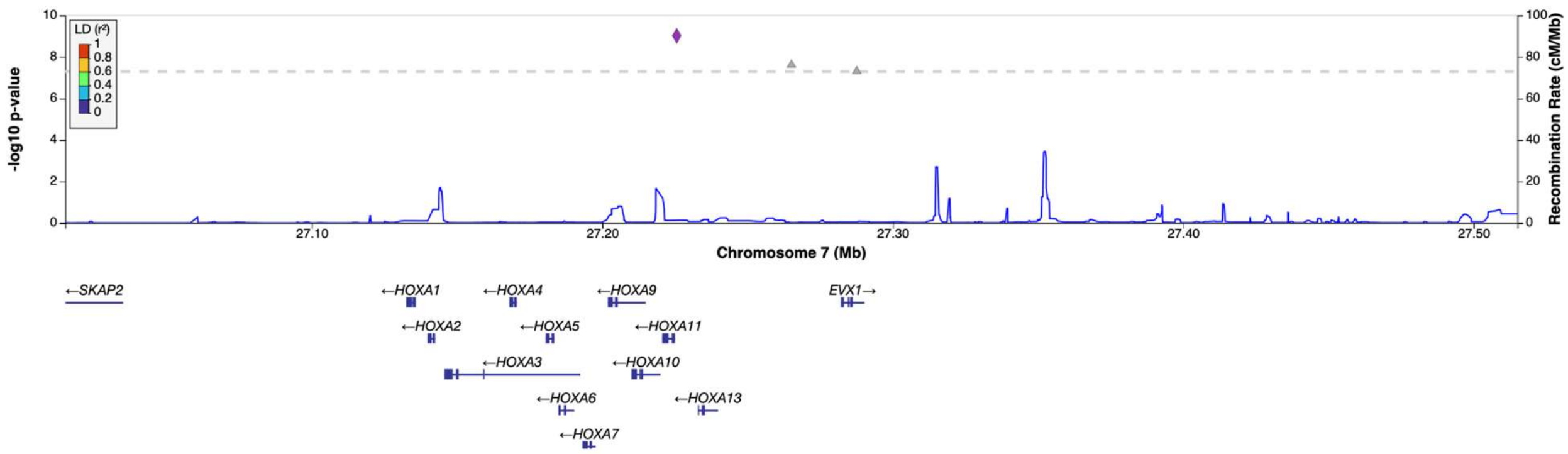

Supplementary Figure S1. Detailed LocusZoom plot for *HOXA11* locus.

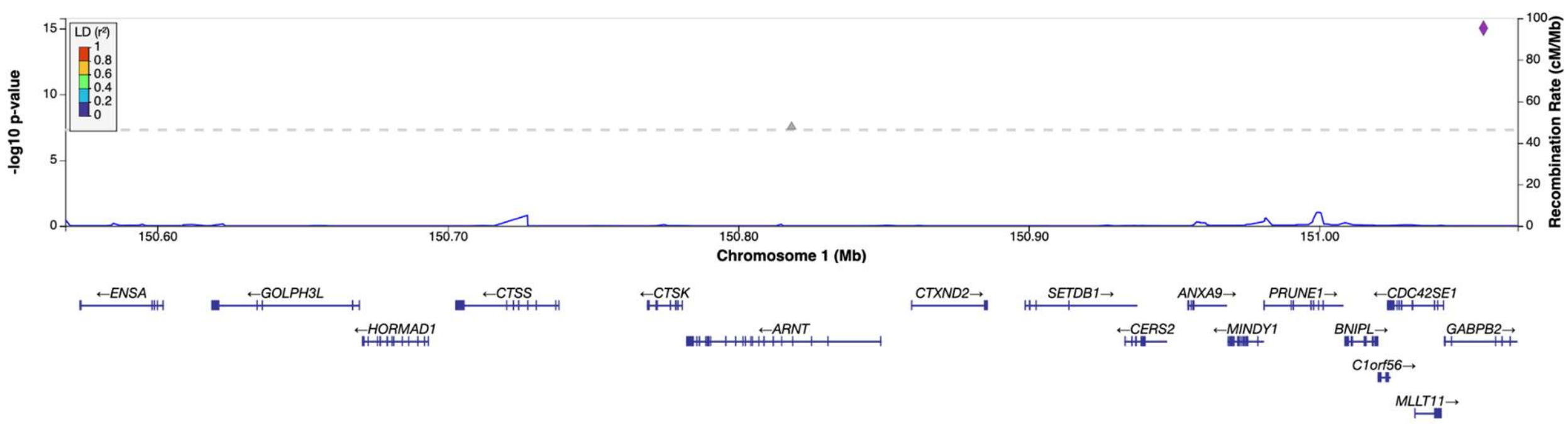

Supplementary Figure S2. Detailed LocusZoom plot for *ARNT* locus.

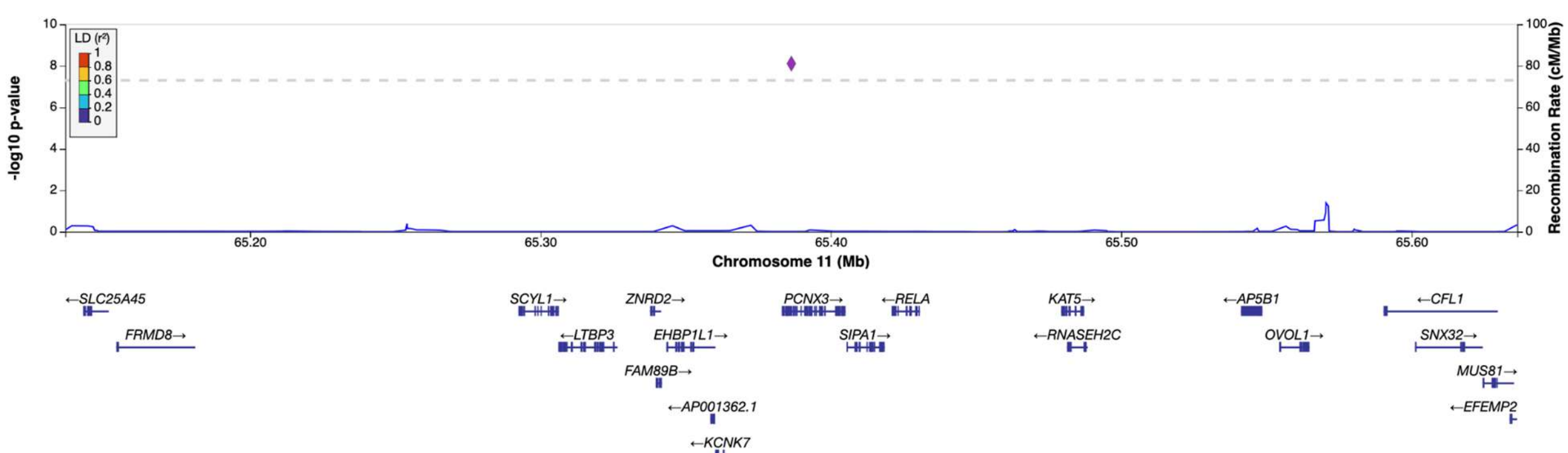

Supplementary Figure S3. Detailed LocusZoom plot for *PCNX3* locus.

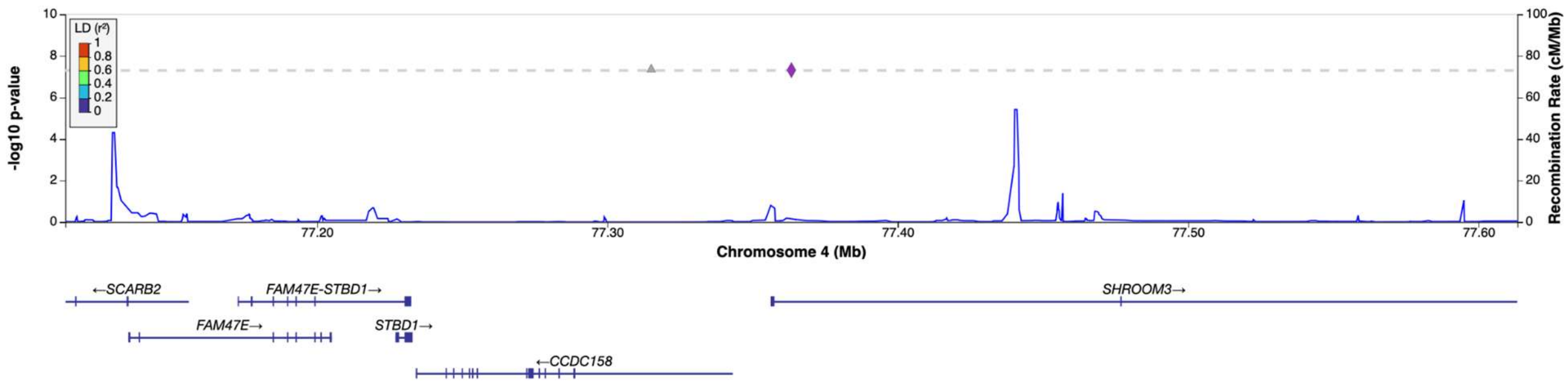

Supplementary Figure S4. Detailed LocusZoom plot for *SHROOM3* locus.

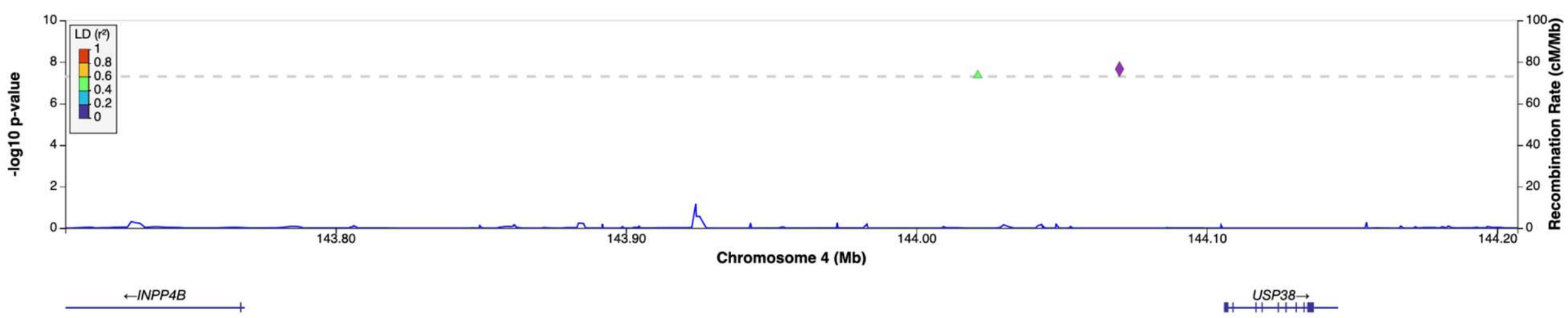

Supplementary Figure S5. Detailed LocusZoom plot for *INPP4B-USP38* locus.

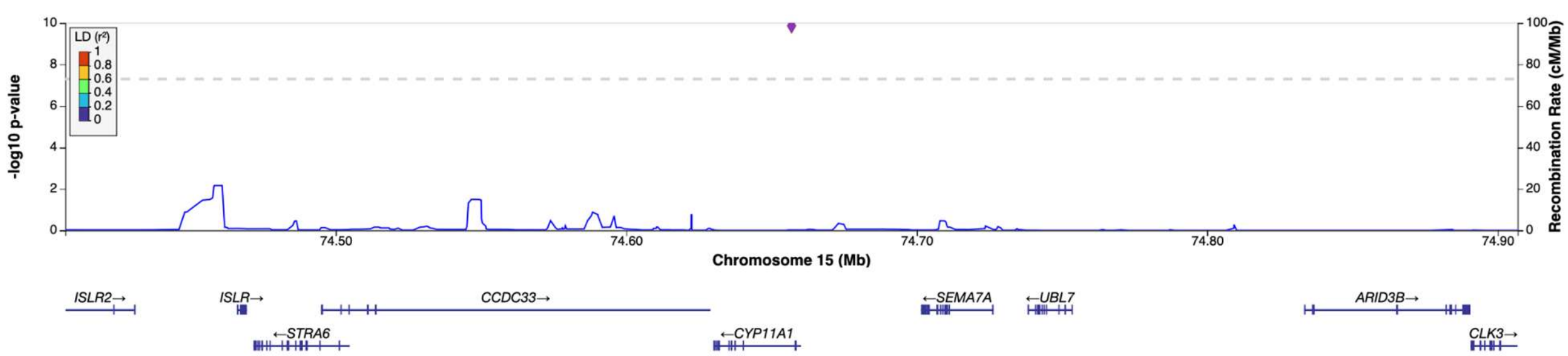

Supplementary Figure S6. Detailed LocusZoom plot for *CYP11A1-SEMA7A* locus.

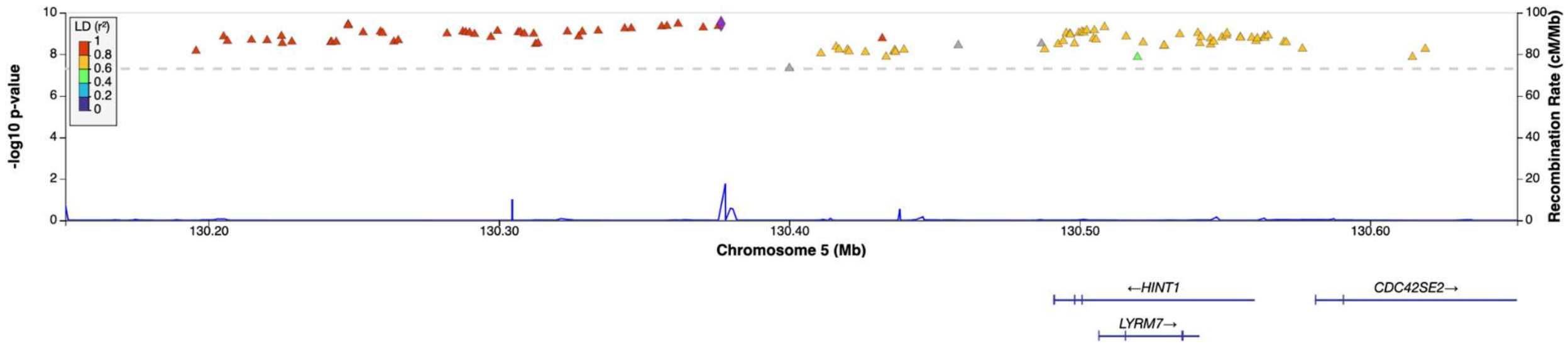

Supplementary Figure S7. Detailed LocusZoom plot for *HINT1* locus.

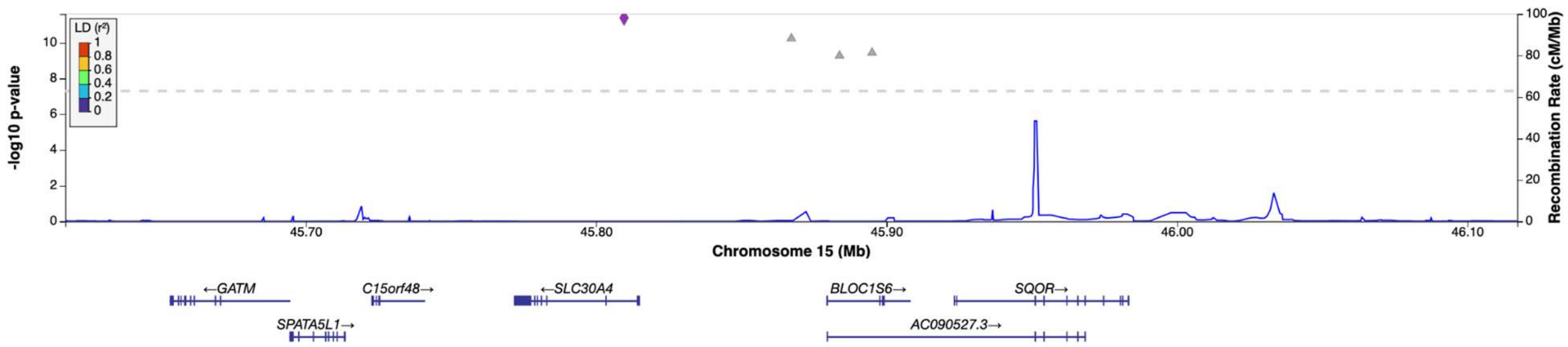

Supplementary Figure S8. Detailed LocusZoom plot for *BLOC1S6* locus.

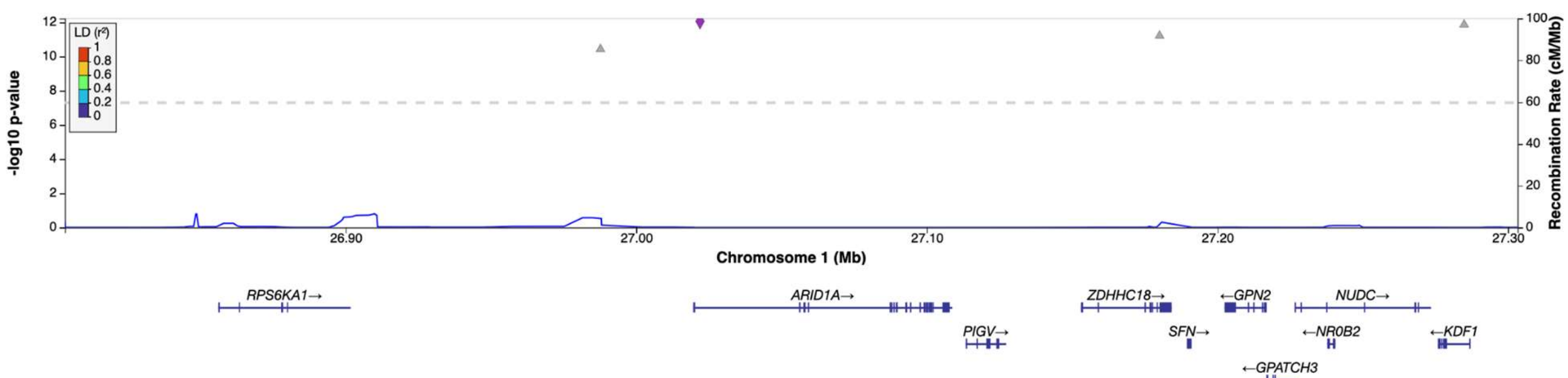

Supplementary Figure S9. Detailed LocusZoom plot for *ARID1A* locus.

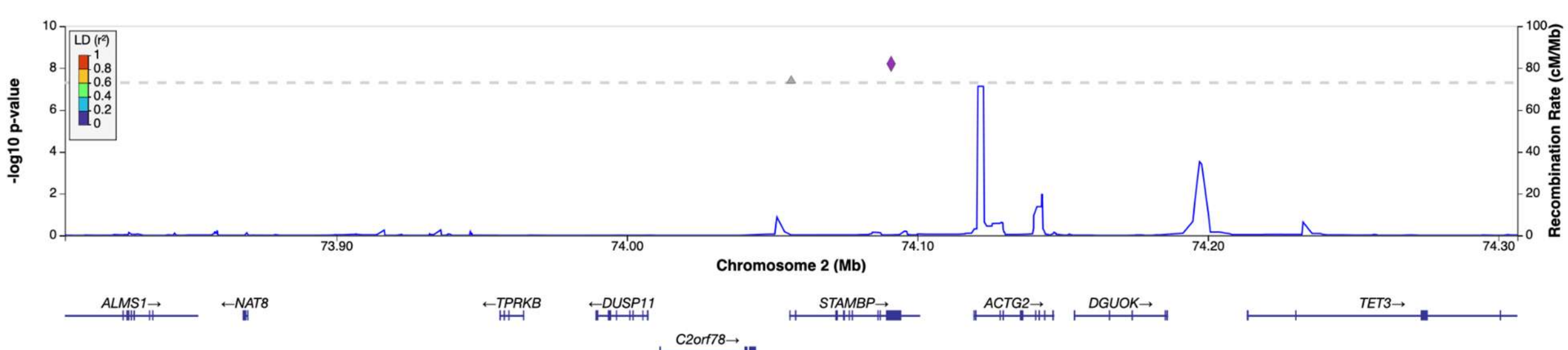

Supplementary Figure S10. Detailed LocusZoom plot for *STAMBP* locus.

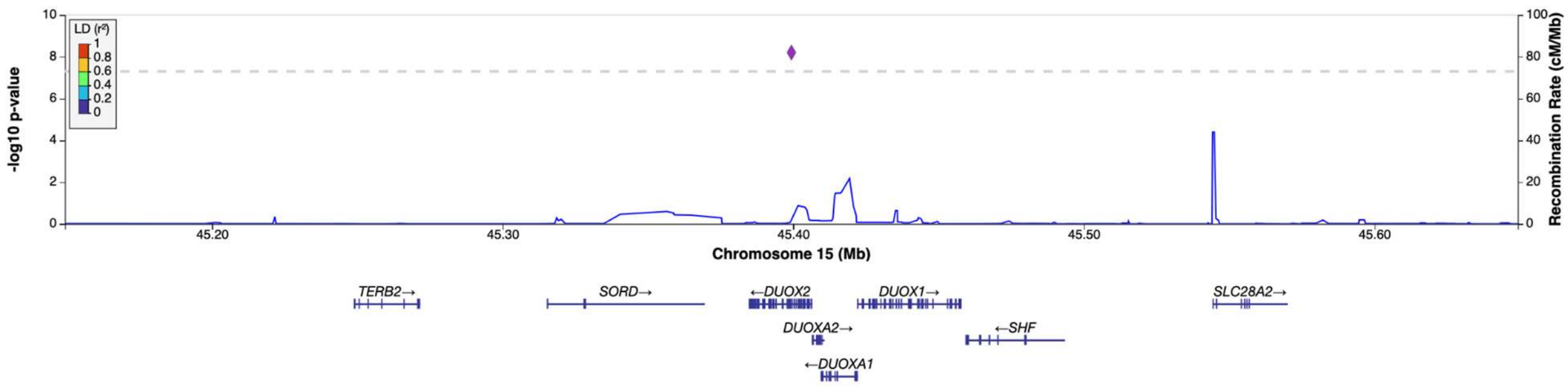

Supplementary Figure S11. Detailed LocusZoom plot for *DUOX2* locus.
